# Within‐patient phylogenetic reconstruction reveals early events in Barrett’s Esophagus

**DOI:** 10.1111/eva.13125

**Published:** 2020-09-20

**Authors:** Lucian P. Smith, Jon A. Yamato, Patricia C. Galipeau, Thomas G. Paulson, Xiaohong Li, Carissa A. Sanchez, Brian J. Reid, Mary K. Kuhner

**Affiliations:** ^1^ Department of Genome Sciences University of Washington Seattle WA USA; ^2^ Division of Human Biology Fred Hutchinson Cancer Research Center Seattle WA USA; ^3^ Department of Medicine University of Washington Seattle WA USA

**Keywords:** ARID1A, Barrett's Esophagus, CDKN2A, esophageal adenocarcinoma, genome doubling, phylogeny, SMARCA4, subclonality, TP53

## Abstract

Barrett's Esophagus is a neoplastic condition which progresses to esophageal adenocarcinoma in 5% of cases. Key events affecting the outcome likely occur before diagnosis of Barrett's and cannot be directly observed; we use phylogenetic analysis to infer such past events. We performed whole‐genome sequencing on 4–6 samples from 40 cancer outcome and 40 noncancer outcome patients with Barrett's Esophagus, and inferred within‐patient phylogenies of deconvoluted clonal lineages. Spatially proximate lineages clustered in the phylogenies, but temporally proximate ones did not. Lineages with inferred loss‐of‐function mutations in both copies of *TP53* and *CDKN2A* showed enhanced spatial spread, whereas lineages with loss‐of‐function mutations in other frequently mutated loci did not. We propose a two‐phase model with expansions of *TP53* and *CKDN2A* mutant lineages during initial growth of the segment, followed by relative stasis. Subsequent to initial expansion, mutations in these loci as well as *ARID1A* and *SMARCA4* may show a local selective advantage but do not expand far: The spatial structure of the Barrett's segment remains stable during surveillance even in patients who go on to cancer. We conclude that the cancer/noncancer outcome is strongly affected by early steps in formation of the Barrett's segment.

## 1 INTRODUCTION

Barrett's Esophagus (BE) is a neoplastic condition in which the normal squamous lining of the lower esophagus is replaced by a columnar epithelium. The clinical description is given by Shaheen et al. (2016). It is associated with gastric reflux disease and smoking, and may be a response to mutagenic damage to the esophagus. BE progresses to esophageal adenocarcinoma (EA) in about 5% of patients; the standard of care involves periodic endoscopic biopsies for early detection of EA. Progression to cancer is associated with lesions in *TP53*, chromosome copy number variation, and genome doubling (GD) (Li et al., 2014). The state of BE research has recently been reviewed by Contino, Vaughan, Whiteman, and Fitzgerald (2017). Very high levels of somatic point mutation are seen in BE and EA but only weakly distinguish cancer outcome (CO) from noncancer outcome (NCO) patients. Table 1 defines abbreviations used in this study.

**TABLE 1:** Abbreviations

BE	Barrett's Esophagus	LOH	loss of heterozygosity
CF	cell fraction	NCO	noncancer outcome
CN	copy number	PPI	proton‐pump inhibitor
CO	cancer outcome	SCA	somatic chromosomal alterations
dN/dS	Nei's selection test	SNP	(germline) single nucleotide polymorphism
EA	esophageal adenocarcinoma	SNV	(somatic) single nucleotide variant
GD	genome doubling	TP1	time point 1 (also TP2, TP3)
GEJ	gastroesophageal junction	U	upper (in the esophagus)
indel	small insertion or deletion	VAF	variant allele frequency
L	lower (in the esophagus)	WGS	whole‐genome sequencing

The development of Barrett's epithelium is poorly understood. It is likely that most BE is not diagnosed until years after it arises, as it is generally asymptomatic; this is supported by a study of changes in methylation decay rates between BE and normal tissue, in which patients with a mean age of 51.2 years at initial diagnosis were inferred to have developed BE at a median age of 33.6 years (Curtius et al., 2016). Indirect evidence suggests that BE arises from residual embryonic stem cells near the gastroesophageal junction (GEJ) and grows upwards (Wang et al., 2011). The segment length observed at initial diagnosis does not tend to increase subsequently, suggesting that growth has generally terminated prior to diagnosis and further implying that the period of growth is brief. Both for general understanding of the condition and for clinical management, it will be important to know when in this process CO and NCO pathways diverge. For example, while proton‐pump inhibitors (PPI) effectively treat gastric reflux symptoms, they have not been markedly successful at reducing risk of EA in patients with BE (Hu et al., 2017): Is this because a cell lineage with cancer‐predisposing mutations (Kuhner, Kostadinov, & Reid, 2016) has already been established before PPI treatment begins?

The companion study shows that there is little change in genomic features associated with progression between the clinical surveillance endoscopy for BE, at the first time point, and subsequent endoscopies several years later. Most CO patients already have biopsies displaying genomic changes associated with progression risk at the initial endoscopy. This suggests that the evolutionary process leading to EA is already well underway before BE is diagnosed, as has also been reported by Martinez et al. (2016) based on a longitudinal study. To understand early events in this process, therefore, we must turn to historical inference rather than direct observation.

Our data set comprises 40 CO and 40 NCO patients with whole‐genome sequencing of 4–6 biopsies per patient from 2–3 time points. In this study, we used phylogenetic analysis of lineages within each patient to probe events during development of the BE segment, and contrast this process in CO and NCO patients. This inference is complicated by the use of bulk‐epithelium data: Each epithelial isolate contains approximately 1 million cells, and these do not always represent a single evolutionary lineage but may arise from multiple subclones. We used a partially automated deconvolution approach to separate the subclones for phylogenetic analysis. This allows exploration of the subclone landscape of BE in CO and NCO patients.

We also examine fourteen loci which had 25+ functional mutations across our data, including four loci that are commonly mutated in BE and EA and show evidence of positive selection. Mutations in *TP53* are strongly associated with chromosomal copy number abnormalities, genome doubling, and progression to cancer (reviewed in Contino et al., 2017). In contrast, mutations and copy number variants in *CDKN2A* are ubiquitous in both CO and NCO, but do not predict progression (Li et al., 2014, ). Mutations in the chromatin‐remodeling genes *ARID1A* and *SMARCA4* are also frequent in BE and EA (reviewed in Contino et al., 2017) and in the companion study were shown to be positively selected in BE; their role, if any, in progression to EA is unclear.

The companion study suggests two separate groups of abnormalities in BE. High somatic single nucleotide variant (SNV) load, somatic chromosomal alterations (SCA) at specific fragile‐site loci, and loss of *CDKN2A* are typical of BE but show little or no association with cancer outcome. Loss of *TP53*, high overall SCA load, structural variants, and genome doubling separate CO from NCO patients, suggesting compromise of cellular mechanisms for ensuring genomic stability. Even in CO patients, many biopsies are indistinguishable from those in NCO; genomically stable and unstable BE lineages can coexist in a patient over a time scale of years. This deviates from the standard model of cancer development as a series of selective sweeps. Using phylogenetic analysis to probe the unobserved early history of the BE segment, we can better understand the natural history of BE and the processes that lead to these two disparate outcomes.

We chose to use a partially automated deconvolution method with significant manual analysis in this study, despite the risk of subjectivity, because in our judgment current fully automated deconvolution techniques do not handle BE data satisfactorily. To simplify the difficult problem of clonal deconvolution, most methods do not permit lineages within a sample to differ in local copy number, and no method we are aware of permits them to differ in overall copy number (ploidy). An initial examination of our data showed that neither assumption could be justified in a substantial number of our patients. In 11/80 patients, both lineages with and without GD were clearly present, and subclonal copy number variants were frequently evident. We have described our manual procedure in some detail so that it can be critiqued. We will be delighted if future improvements in deconvolution software allow a fully automated approach, but until then, we believe that the approach described here has the best chance of extracting useful information from the complexity of bulk‐sequenced epithelium.

## 2 METHODS

### 2.1 Patients and samples

Data were obtained from 40 CO and 40 NCO patients diagnosed with BE, taken from a larger case‐cohort study (Li et al., 2014) within the Seattle Barrett's Esophagus Program at the Fred Hutchinson Cancer Research Center. All research participants contributing clinical data and biospecimens to this study provided written informed consent, subject to oversight by the Fred Hutchinson Cancer Research Center IRB Committee D (Reg ID 5619).

Cohort characteristics and data collection are described in the companion study. Briefly, cancer outcome (CO) was defined as a histologic diagnosis of EA during surveillance, and noncancer outcome (NCO) as absence of such diagnosis, regardless of dysplasia grade. Two samples were analyzed per patient per time point (TP), with 2 TP in CO patients and 2 or 3 in NCO patients. Among 40 CO patients, for 38 the TP2 endoscopy was the endoscopy at which EA was detected, with a mean of 2.9 years between TP1 and TP2. For reasons of sample availability, for the remaining two patients the TP2 endoscopy was the endoscopy previous to detection of EA, with a mean of 2.5 years TP1‐TP2 and 2.9 years TP1‐EA. The 40 NCO patients included 30 patients with 2 time points, with a mean of 3.4 years between TP1 and TP2, and a mean of 7.7 years from TP1 to the last endoscopy on record; and 10 long‐followup patients with a mean of 3.8 years between TP1 and TP2, and a mean of 13.2 years between TP1 and TP3, which was the last endoscopy on record. NCO were matched to CO based on baseline total somatic chromosomal abnormalities (SCA) as measured in a previous study of the cohort (Li et al., 2014); age at TP1; and time between TP1 and TP2. It should be noted that matching on SCA biased the NCO group toward higher levels of SCA (possibly indicating a more severe condition) than would be typical of a random sample of NCO patients, reducing the chance of detecting differences between CO and NCO. We found little difference between preliminary analyses including or excluding the TP3 samples, so have included them throughout this study except where specified otherwise. Sample‐level dysplasia grade was not available for the sequenced samples and thus was not considered in the analysis.

### 2.2 Sequencing

Epithelial isolation was used to separate the BE epithelium from the underlying stromal tissue, generating samples which were >98% pure epithelium (Li et al., 2014). Samples were whole‐genome sequenced to 60x and matched blood or gastric controls to 30x. Each sample was also run on a 2.5M SNP array for copy number analysis. Details of sequencing and mutation calling are presented in Paulson et al. (in preparation). Briefly, muTect (Cibulskis et al., 2013), Strelka (Saunders et al., 2012), and LoFreq (Wilm et al., 2012) were used to call somatic SNVs; only SNVs detected by at least 2 of 3 callers were retained.

The sequencing and SNP array data are under embargo until September 30, 2020, and will subsequently be available at: http://www.ncbi.nlm.nih.gov/projects/gap/cgi-bin/study.cgi?study_id=phs001912.v1.p1

### 2.3 Copy number and ploidy calling

Ploidy of samples was assigned by hand, as we have found that automated ploidy calling overcalls tetraploids in situations with high subclonality compared to expert hand assignment. Ploidy calls were made by consensus of the research group based on WGS and SNP array data, supplemented with SNP array and DNA content flow cytometric data previously obtained from other samples from the same patients. The *pASCAT* program (described in Smith, Yamato, & Kuhner, 2019; see Software Availability section at end of methods), a modification of *ASCAT* (Van Loo, Nordgard, Lingjærde, Russnes, & Rye, 2010), was used to assign local allele‐specific copy number calls based on 2.5M SNP array data. Depending on the assessed ploidy of the sample, either a *pASCAT* solution constrained to be near‐diploid (1.0–2.9) or a solution constrained to be near‐tetraploid (2.5–6.0) was used. Assessments of overall copy number calling accuracy for diploid and tetraploid solutions were made using the *CNValidator* program (Smith et al., 2019).

### 2.4 Deconvolution algorithm

Use of existing deconvolution software was challenging due to allelic dropout, copy number variation, and ploidy variation. We therefore used a semi‐automated hand inference procedure. The algorithm is given in detail in Appendix A, and more briefly here.

Within‐patient phylogenies were drawn based only on somatic SNVs; indels were less frequent and more challenging to interpret, so were not used in phylogeny construction. In our data over 99% of SNVs were given a severity score of MODIFIER or LOW by SnpEff (Cingolani et al., 2012) and were therefore likely evolutionarily neutral. We therefore assumed that averages over large numbers of SNVs would represent the evolutionary trajectory of the clone containing them, and not selective effects on the individual SNVs.

For each patient, we separated SNVs into partitions based on the subset of samples in which they occurred. Within each sample, we further subdivided each partition into groups with the same local copy number call. For each group containing at least 100 SNVs, we estimated one or more mean variant allele frequencies (VAFs, defined as mutant reads/total reads), using custom kernel‐smoothing code to locate peaks in the histogram of VAF values, and the *pymix* library (Georgi, Costa, & Schliep, 2010), which estimates mixtures of Gaussians, to estimate the number of sites in each peak. We discarded peaks corresponding to fewer than 100 SNVs, and discarded groups entirely if no peak had 100+ SNVs. We also discarded SNVs for which one or more samples lacking the SNV had a copy number call indicating loss of one haplotype at its position (deletion, copy‐neutral LOH) as the ancestral presence or absence of the SNV could not be reliably determined. Three biopsies which had fewer than 100 SNVs in any partition were dropped from the phylogenetic analysis. These biopsies may represent inadvertent sampling of non‐BE tissue; in any case, they lacked sufficient signal for phylogenetic analysis.

We then heuristically assembled a phylogenetic tree, along with estimates of cell fraction (CF). We began with the partitions containing exactly two samples and worked toward the root; if two partitions contained the same number of samples, we resolved the partition with the larger number of SNVs first. To add a new partition to the phylogeny, we noted whether any of the samples in the partition were already present. If none were, the partition was added as a new clade in the phylogeny. If one or more samples were already present, we inspected the tree to see if there was a better fit for treating the new partition as extending previous clades, or establishing new ones. When, for example, SNVs were present for a partition of samples A, B, and C, but not for any pairwise combination of these, we drew the phylogeny as an ABC polytomy to indicate uncertainty.

**FIGURE A2:**
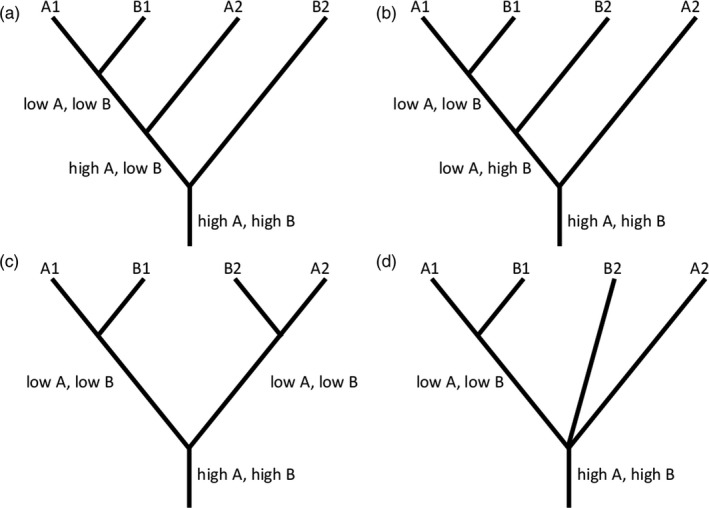
Resolution of ambiguity when both lineages in a partition show split VAF*2 values. Panel a, resolution in which B2 is the outgroup. Mutations arising on the indicated branch should have high VAF*2 in sample A and low VAF*2 in sample B, whereas mutations with the opposite pattern should not arise on this resolution. Panel b, resolution in which A2 is the outgroup, showing the opposite situation. Panel c, a third possible resolution which does not generate a high/low pattern for any SNVs. Panel d, ambiguous (polytomy) phylogeny used when the SNV pattern could not distinguish between the situations in panels a and b

When two or more VAF peaks were present for a partition, we considered multiple hypotheses. Chromosomal regions with an unbalanced copy number (CN) naturally generate multiple VAF peaks, but we allowed this explanation only when *pASCAT* had called an unbalanced CN. Genome doubling occurring on a phylogenetic branch can generate two peaks differing by a factor of two in all samples descending from that branch. When we observed this factor‐of‐two relationship in samples already determined to be genome doubled by the analysis of Paulson et al. (in preparation), we used this interpretation in phylogeny construction. In two patients, a pattern resembling genome doubling was observed but there was no external corroboration; we did not treat this as genome doubling. The third explanation for multiple peaks is that the partition represents two different lineages, both relating the same samples but at different time depths, and we split the sample accordingly (see Figure A2 in Appendix A).

We did not make connections among lineages unless we observed 100+ shared SNVs. As a result, some “phylogenies” consisted of two, three, or four disjoint trees. These may represent independent origins of BE from healthy tissue, or a single origin with early branching before mutations could accumulate.

We assigned the CF of each tip in the phylogeny by averaging estimates from all relevant partitions, weighting by the number of SNVs in each partition. Mutations from areas of unbalanced copy number were assigned to the haplotype most consistent with the CF seen in areas of balanced copy number, and their VAF was interpreted based on the inferred copy number of that haplotype.

We did not attempt to deconvolute private mutations; we treated a tip lineage as having the CF it possessed when diverging from the rest of the phylogeny, even if its private‐mutation VAFs suggested a much lower CF.

In some cases, the set of partitions was not logically compatible with any tree. In most cases, this could be resolved by treating an infrequent partition as representing allelic dropout of a more frequent one, with SNVs corresponding to low‐CF lineages being missed in sequencing.

Each deconvolution was done twice; if the two resolutions did not agree, the deconvolution team (LPS, JAY, MKK) examined them and attempted to come to a consensus. In two CO patients (#74 and #396), no solution could be found; these patients are omitted from all analyses. Examination of these patients suggested high subclonality with ploidy varying among subclones in the same biopsy, and consequent incoherence of the copy number calls, as assessed with the validation tool *CNValidator* (Smith et al., 2019). Omission of these particularly genomically unstable patients is likely to bias estimates of GD, *TP53* mutation, and subclonality downwards and to make CO and NCO appear more similar, but we were unable to find a viable alternative. We thus had a final data set of 329 biopsies out of the original 340, omitting eight biopsies from the two unresolvable patients and three biopsies that had less than 100 total SNVs.

Deconvoluted phylogenies are archived on Dryad: doi: 10.5061/dryad.866t1g1p1.

### 2.5 Assigning mutations to branches

An automated algorithm was used to assign SNVs to internal branches of the trees to determine branch lengths. For the majority of SNVs, this was straightforward: An SNV seen in multiple samples was assigned to the branch that led to tip lineages seen in exactly those samples. SNVs which could not be assigned to any branch in this way (they were discordant with the inferred phylogeny) were dropped from analysis.

In some cases, multiple branches in the tree led to tip lineages seen in the same group of samples. In those cases, the VAF of the SNV was used to select its branch. The Software Availability section describes availability of code to perform these assignments.

### 2.6 Proportion of SNVs used in tree building

SNVs potentially useful for phylogeny inference excluded those which fell in deleted regions, displayed more than 2 alleles, or were private to a single sample. We assessed what proportion of the remaining SNVs were placed on the phylogeny by our analysis. The mean over patients was 94.5%; the median was 96.1%; and the minimum (seen in a patient with large amounts of copy number variation) was 72.1%. We are therefore confident that our phylogenies are broadly supported by the SNV data.

### 2.7 Simulation‐based significance testing

The process which generates within‐patient phylogenies is difficult to model statistically, as key parameters are unknown. We therefore used a strategy of permuting or resampling data on the actual inferred phylogenies for significance testing. The general procedure was to generate 10,000 resampled data sets and score what proportion of these data sets produced an estimate more extreme than the estimate from the actual data. Ties between simulated and actual data were resolved randomly, and for a two‐tailed test, the inferred tail fraction was doubled. When events (candidate‐locus mutations, candidate‐locus losses of heterozygosity, genome doublings) were randomly resimulated on branches of trees, we used the inferred branch length (based on SNVs) to weight resimulation. When states were permuted among branches of trees, we constrained tips derived from the same biopsy to have the same state in the resimulated data (e.g., all tips derived from the same biopsy must share the same spatial coordinates).

For cases in which multiple loci or locus pairs were being individually evaluated for significance, we report both uncorrected *p* values and *q* values based on the Benjamini‐Hochberg multiple testing correction (Benjamini & Hochberg, 1995) as implemented in the Python library *statsmodels* (Seabold & Perktold, 2010).

### 2.8 Topology analyses

Preliminary analysis using parsimony trees of bulk‐epithelium data without deconvolution () suggested that biopsies which fell in the same general region of the BE segment were clustered on the phylogenies, whereas there was no clustering of biopsies from the same time point. We extended this analysis to use deconvoluted trees.

For time analysis, we classified each biopsy by time point (TP1, TP2, or TP3). To classify biopsies for spatial analysis, we calculated each biopsy's vertical position in the esophagus relative to the gastroesophageal junction (GEJ: the lower boundary of the BE segment) and identified the lowest and highest biopsies. One patient was excluded from this analysis as all four biopsies were at the same distance from the GEJ. The lowest biopsy, and any biopsies closer to it than to the highest, were assigned code “L” (“lower”); remaining biopsies were assigned code “U” (“upper”). Biopsies equidistant between lowest and highest were assigned to the less numerous group, or arbitrarily to “U” if the groups were equal in size.

To assess clustering, we used the minimum number of changes of state (e.g., L to U or U to L) required by the deconvoluted phylogeny as a measure of its clustering. We compared this with resimulated data which permuted the assignment of U/L among biopsies. We used a one‐tailed significance test, as our expectation was that biopsies from the same region or time point would be clustered, not dispersed.

We also considered a biopsy's distance from the GEJ in centimeters as an ordered trait and did ordered‐trait parsimony to assess clustering, testing significance via the permutation approach described above.

In some patients, the deconvoluted lineages fell into two or more disjoint trees, presumably representing either multiple independent origins of BE, or an original BE lineage which lacked substantial mutations. We did not score any changes of state between disjoint trees. Thus, a patient with two L and two U biopsies could have a score of 0 state changes if their biopsies came from multiple unrelated lineages, whereas at least one state change would be needed if all biopsies arose from the same lineage. It is not known where BE originates, though it is hypothesized to be near the GEJ (Wang et al., 2011); however, we have not assumed that the ancestral lineage on our trees is GEJ‐proximal as a downwards selective sweep could invert the original relationships.

For each scoring criterion (time point, U/L classification, cm from GEJ), we tested for difference between real and resimulated data in all patients, CO only, and NCO only.

We defined a sample as subclonal if it was represented by two or more lineages in the phylogeny, disregarding evidence of subclonality in private SNVs. We tested for per‐patient clustering of subclonality (was the number of patients with subclonal samples greater or less than would be expected given the total number of subclonal samples?) by permuting the subclonality status of samples across the entire data set and scoring the number of patients with one or more subclonal samples. We also tested whether subclonal samples were disproportionately present in CO or NCO patients, in U or L locations, or in TP1 or TP2 using a similar permutation approach. For the last analysis, the 20 TP3 samples were omitted.

### 2.9 Analysis of specific loci

The effects of mutations were assessed using SnpEff (Cingolani et al., 2012). Functional mutations were defined as those with SnpEff severity MODERATE (missense) or HIGH (nonsense, frameshift, splice site).

We defined candidate loci for this test as those with 25 or more functional mutations across our data set. There were 14 such loci: *ARID1A, CDKN2A, CSMD1, CSMD3, FAT3, LAMA1, LRP1B, MUC16, MUC19, PCLO, SMARCA4, SYNE1, TP53,* and *TTN*. Of these, *TP53* and *CDKN2A* have been repeatedly associated with BE in previous publications (Li et al., 2014; Stachler et al., 2015); those two loci as well as *ARID1A* and *SMARCA4* showed a significant excess of functional mutations in this data set using dN/dS in the companion study. Conversely, *TTN* is generally considered a false positive in cancer analysis as its high mutation frequency can be attributed to its inordinate size. We plotted inheritance of functional SNVs in these loci on our deconvoluted phylogenies, and compared them with the overall distribution of SNVs on phylogeny branches, a distribution dominated by the 99% of all SNVs assessed to be nonfunctional.

Candidate‐locus SNVs were assigned to phylogeny branches by hand, rather than using the general algorithm for assignment of SNVs to branches. Hand analysis was required because mutations at several of these loci (*TP53* and *CDKN2A* in particular) tended to be homozygous and to lie in areas of abnormal copy number, complicating the analysis. We also hand‐assigned homozygosity or heterozygosity (defined as having no copies or at least one copy of the wild‐type allele, respectively) of each mutation by computing the expected VAF of a homozygous or heterozygous mutation on a lineage of the given CF, and choosing the numerically closer solution. When assignment of the mutation to a particular branch allowed the mutation to be heterozygous, and assigning it to a different branch forced it to be homozygous, we chose the heterozygous solution. Candidate‐locus mutations in the two patients who could not be deconvoluted were dropped from analysis.

A limitation of this analysis is that, because we were not able to reliably assign CN variants to subclones, loss of function of a candidate gene locus due purely to deletion was not detected. Losses of *CDKN2A* will be particularly underestimated by our methods as this fragile‐site locus is frequently deleted in BE and EA (Li et al., 2014). We expect this issue to reduce statistical power for comparing *CDKN2A* mutant lineages to wild‐type ones, as some proportion of putative wild‐type lineages will carry a single or double deletion at *CDKN2A*, but we do not expect it to otherwise bias the results.

To assess expansion of clones carrying candidate mutations, we computed the total number of phylogeny leaves, and the total estimated CF of these leaves, for lineages carrying mutations in each locus across all patients. Significance was determined by comparing these data with simulated data which placed the same number of candidate‐locus mutations randomly onto branches, weighted by inferred branch lengths. Two‐tailed tests were used as we could not be sure a priori whether possession of a candidate mutation would increase or decrease spread of the lineage.

We repeated this analysis separating mutations inferred to be homozygous or heterozygous. In a handful of patients, the same mutation was seen to be homozygous in some lineages and heterozygous in others. We assumed that the ancestral state was heterozygous and that the mutation had become homozygous in subsequent events, and scored branches accordingly. In the simulated data, we chose an initial mutation from among the branches which would permit the needed number of secondary changes, and then chose the secondary changes among branches tipward of the initial mutation.

We tested whether candidate mutations were correlated or anti‐correlated across patients (i.e., whether a patient with one mutation in a candidate locus was more or less likely to have additional mutations in the same locus, relative to their overall SNV count) by counting the candidate mutations and then creating resampled data sets where that number of mutations were chosen at random across all patients, proportional to the inferred branch lengths. We used a two‐tailed test.

We also tested for ordering of mutations in pairs of candidate loci, and ordering of GD with regard to the candidate loci. For a patient with mutations in two loci A and B, there are four possible configurations: both on the same branch, mutation A in a branch ancestral to mutation B, mutation B in a branch ancestral to mutation A, and mutations A and B on skew branches. We scored these configurations in the real data and in simulated data sets made by taking each patient with two or more candidate mutations and randomly sampling new locations for these mutations, weighted by inferred branch lengths. Values from a single patient were averaged; this avoids potential bias if, for example, a patient had an early mutation in one locus and multiple mutations in the other locus with which to compare it. We used a chi‐square test for heterogeneity with 6 *df* to compare the real data with the simulated data.

### 2.10 Software availability

pASCAT: https://github.com/kuhnerlab/pASCAT

Deconvolution scripts:

https://github.com/kuhnerlab/subclone_deconvolution

## 3 RESULTS

### 3.1 Many biopsies contain phylogenetically distinct subclones

Out of 329 biopsies, 145 were inferred to be comprised of two or more distinct lineages. Note that these counts exclude cases in which tip lineages split further after divergence from the rest of the phylogeny, as we were not able to accurately deconvolute these. Subclonal biopsies were significantly clustered among patients (*p* = .0153, two‐tailed test): A subset of patients contained a disproportionate share of the subclonal biopsies, suggesting that lineages were intermixed more freely in some patients than others.

While both CO and NCO had extensive subclonality, there were more subclonal biopsies in NCO relative to expectations (*p* = .0046, *q* = 0.0139, two‐tailed test). However, this result may be due to greater power to detect subclonality in patients with a larger number of biopsies (10 NCO patients had six rather than four biopsies) as the comparison was no longer significant when only TP1 and TP2 biopsies were used (*p* = .0861, *q* = 0.2582). There was no excess of subclonality in upper versus lower biopsies (*p* = .3652, *q* = 0.4177) or in TP1 versus TP2 (*p* = .4711, *q* = 0.4711). In these data the companion study showed that CO have slightly more unique mutations per patient than NCO; the results here show that this is not due to larger numbers of distinct lineages, but instead likely arises from a difference in mutation rate or clonal spread (which affects the proportion of mutations abundant enough to be detected).

### 3.2 Independent origins of BE

Several studies have found either multiple BE clones (Ross‐Innes et al., 2015) or BE and adjacent EA (Stachler et al., 2015) that share very few mutations, suggesting possible independent origins from wild‐type tissue. We defined lineages as representing separate origins if they shared fewer than 100 SNVs, excluding from consideration biopsies in which no partition (including private SNVs) had more than 100 SNVs, as it is not clear that such biopsies represent BE tissue. The cutoff of 100 SNVs was chosen because partitions of fewer SNVs did not seem phylogenetically reliable; any two biopsies in a patient are likely to share a handful of SNVs due to a combination of sequencing errors, errors in the normal reference sequence, allelic dropout, and convergent evolution.

**FIGURE 1:**
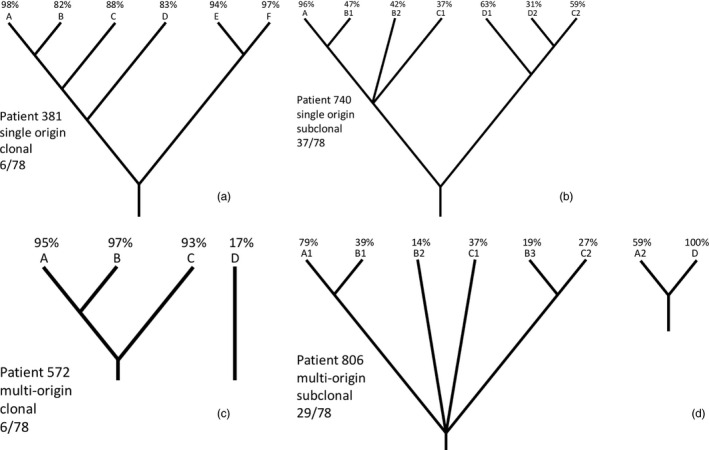
Patient phylogenies with single and multiple origins, with and without subclonality. A representative phylogeny with high deconvolution confidence is shown for each category; the fraction beneath it indicates what proportion of patients fell into this category. Diagram branch lengths are arbitrary. In tip labels, letters indicate biopsy, following numbers distinguish multiple lineages in the same biopsy, and percentages are inferred cell fractions (CF). The CF values for a given biopsy may sum to less than 100%, likely representing failure to detect minority clones

By these criteria, 43/78 (55%) of patients had a single origin for all samples (Figure 1 panels a,b) and 35/78 (45%) of patients had multiple origins (Figure 1 panels c,d): 29 with two origins, 5 with three, 1 with four. The frequency of multiple origins did not vary between CO and NCO (*p* = .25, ns). We reran the analysis using only TP1 and TP2 samples to avoid any bias by the larger number of samples in NCO, but the results were nearly identical (33/78 or 42% had multiple origins and CO and NCO did not differ significantly). Of note, in several patients all samples shared detectable SNVs, and previous methods would have inferred a single origin, but deconvolution revealed multiple independent lineages (see Appendix B for an example). As we examined only 4–6 biopsies per patient, and could not reliably detect subclones smaller than approximately 15% of cells in a biopsy, our estimate of origins per patient is likely to be an underestimate. We cannot determine from these data whether the common ancestor of the sampled biopsies was phenotypically neoplastic or carried epigenetic changes, but in many patients it appears to have lacked the typical high SNV mutation rate of fully developed BE. Furthermore, lineages of apparently separate origin are sometimes intermixed in a single biopsy.

### 3.3 Biopsies are related in space, but not time

**FIGURE 2:**
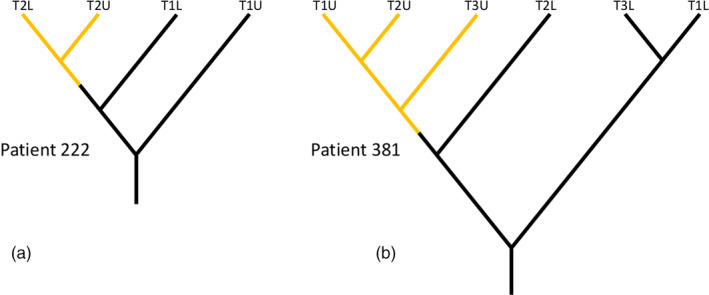
Spatial and temporal clustering. Panel a: Patient 222, displaying temporal clustering: Samples from T2 (time point 2) form a group, while upper (U) and lower (L) do not. Panel b: Patient 381, displaying spatial clustering: Upper samples form a group, while time points are mixed throughout the tree. While individual patients displayed both patterns as well as inconclusive ones, in the full data set there was an excess of spatial clustering over expectations, but no excess of temporal clustering. Lengths of branches in diagrams are arbitrary

Biopsies from the same region of the esophagus clustered in the deconvoluted phylogenies more often than expected by chance, as shown in Table 2. This effect was present in both CO and NCO patients but more pronounced in CO. No such clustering was seen between biopsies from the same time point. Figure 2 illustrates the patterns associated with spatial or temporal clustering with patient phylogenies: Statistically, temporal clustering was seen about as often as expected by chance, and spatial clustering significantly more frequently.

**TABLE 2:** Phylogenetic clustering of biopsies in time and space

Criterion	All patients	CO	NCO
Time Point	0.2368 (0.2664)	0.1284 (0.1651)	0.4354 (0.4354)
Upper/Lower	**0.0005 (0.0015)**	**0.0005 (0.0015)**	0.0552 (0.0827)
cm from GEJ	**0.0002 (0.0015)**	**0.0099 (0.0178)**	**0.0029 (0.0065)**

Degree of phylogenetic clustering in the observed data is compared with clustering in data with permuted labels. Values in table are uncorrected *p* values (Benjamini‐Hochberg corrected *q* values in parenthesis). Boldface indicates *q* value below 0.05.

### 3.4 Multiple occurence of GD

Fifteen patients had one or more biopsies inferred to contain a GD lineage. Two of these patients could not be deconvoluted. Among the remaining patients, 4/13 showed evidence of GD events in two separate lineages. These could be coincidental, symptomatic of a GD‐prone environment, or represent an ancestral lineage with a propensity to undergo GD.

### 3.5 Analysis of candidate loci

Throughout this section, only functional SNVs (SnpEff MODERATE or HIGH) in the 14 loci with 25+ such SNVs were considered.

If the genomic or environmental background of a specific patient generates particularly strong selection for mutations in a specific locus, such mutations may be concentrated in a subset of patients. Conversely, if the first mutation is selectively favored but additional ones are not, mutations may be dispersed, with fewer patients having multiple mutations than expected. After correction for multiple comparisons, neither effect was significant for any locus.

Table 3 presents tests of propagation of candidate mutations (and GD events) by phylogeny leaf and cell fraction. There was a clear signal for loci *TP53* and *CDKN2A*: mutations in both loci were propagated to significantly more leaves and more cells than random SNVs, and for both loci, this effect was driven by lineages in which the mutation was inferred to be homozygous (i.e., no wild‐type copies were present). The only other significant result was a lowered cell fraction for homozygous mutations in PCLO, suggesting that they are detrimental. This does not rule out detrimental effects of mutations in other loci: A strongly detrimental mutation will neverbe seen in data of this kind as all, as it would have to expand to many thousands of cells in order to be detected.

**TABLE 3:** Expansion of candidate‐locus mutations

Locus	Metric	Overall (sim)	*p*‐value (*q*‐value)	Het. (sim)	*p*‐value (*q*‐value)	Hom. (sim)	*p*‐value (*q*‐value)
*CDKN2A*	Leaves	98.0 (48.7)	**<0.0001 (<0.0001)**	44.0 (31.7)	0.114 (0.599)	54.0 (31.6)	**<0.0001 (<0.0001)**
	Cell fraction	5,101.6 (2,746.7)	**<0.0001 (<0.0001)**	2,015.8 (1,709.9)	0.378 (0.706)	3,085.8 (1,755.0)	**<0.0001 (<0.0001)**
*TP53*	Leaves	119.0 (86.1)	**<0.0001 (<0.0001)**	48.0 (41.4)	0.274 (0.599)	71.0 (49.8)	0.004 (0.056)
	Cell fraction	7,908.8 (5,428.3)	**<0.0001 (<0.0001)**	3,167.8 (2,531.9)	0.090 (0.400)	4,740.9 (3,219.6)	**<0.0001 (<0.0001)**
*PCLO*	Leaves	52.0 (54.7)	0.748 (0.972)	50.0 (54.7)	0.612 (0.917)	2.0 (3.3)	0.150 (0.599)
	Cell fraction	3,112.6 (3,272.1)	0.758 (0.966)	3,082.6 (3,296.3)	0.652 (0.869)	30.0 (244.0)	**<0.0001 (<0.0001)**

Leaf count and cell fraction of candidate‐locus functional mutations are compared to random mutations. Rows marked “leaves” give total number of leaves (tips of the phylogeny) descending from lineages with mutations in the given locus (all mutations in column “Overall,” only homozygous or heterozygous mutations in the corresponding columns). Rows marked “cell fraction” give the sum of inferred CF values for those leaves. In these columns, numbers in parenthesis indicate means of randomized data. Significance is assessed with a two‐tailed test; first number is raw *p* value, value in parentheses is Benjamini‐Hochberg corrected *q* value. Boldface indicates entries with *q* < 0.05

These results contrast with the companion study's detection of positive selection by *dNdScv* (Martincorena et al., 2017) for loci *ARID1A* and *SMARCA4*in the same data set. Both CF and number of leaves were actually depressed for *SMARCA4* mutations, although this was not significant after correction for multiple comparisons. The reason for this discrepancy is unclear. It may reflect selection in different directions during different stages of BE development (for example initial survival versus long‐term spread).

Lineages inferred to contain a GD event did not yield more leaves or cells than lineages with a randomly selected SNV, although this comparison must be treated with caution as it implicitly assumes the frequency of SNVs on a lineage is a proxy for the frequency of GD.

For each patient who had mutations in two or more of these loci, or a mutation and GD, we scored whether the events occurred on the same branch, one was ancestral to the other, or they were on skew lineages. Locus pairs for which the corrected significance was less than 0.05 are shown in Table 4.

**TABLE 4:** Phylogenetic ordering of mutations and GD

Locus 1	Locus 2	# Patients with both mutations	*p* value	*q* value	Most elevated class
*CDKN2A*	*CSMD1*	9	0.001	0.010	*CDKN2A* first
	*MUC16*	11	2.76 × 10^−6^	9.52 × 10^−5^	*CDKN2A* first
	*SMARCA4*	8	8.13 × 10^−9^	8.53 × 10^−7^	*CDKN2A* first
	*SYNE1*	8	1.03 × 10^−5^	1.81 × 10^−4^	*CDKN2A* first
	*TTN*	18	4.53 × 10^−6^	9.52 × 10^−5^	*CDKN2A* first
*TP53*	*MUC16*	19	4.02 × 10^−4^	0.005	*TP53* first
	*TTN*	31	5.75 × 10^−5^	8.63 × 10^−4^	*TP53* first
	GD	14	6.02 × 10^−4^	0.007	*TP53* first

Only pairs for which *q* < 0.05 are shown. Significance values come from an overall chi‐square test for heterogeneity (6 *df*) between actual and simulated results. The p values are uncorrected; q values are corrected for multiple comparisons via the Benjamini‐Hochberg algorithm. The “Most elevated class” column indicates which of the four categories (locus 1 first, locus 2 first, same branch, skew branches) was most elevated over the simulation expectations

*CDKN2A* mutations were disproportionately on lineages ancestral to mutations in other loci: 10/13 loci and GD had *p* < .05 for this relationship, and for 5 loci, this effect was significant after multiple test correction (shown in Table 4). Because the same effect is essentially seen with every comparison, we do not believe it indicates any special relationship between *CDKN2A* and the other loci. Instead, it likely represents a tendency for *CDKN2A* mutations to arise early and spread widely, as shown by the analysis of Table 3, making it likely that randomly occurring mutations in other loci will fall in lineages already mutant for *CDKN2A*.

In contrast, *TP53* mutations had a strong tendency to be ancestral to mutations in *MUC16* and *TTN*, and to GD, but not other loci. *TP53* mutations likely contribute to the development of GD (e.g., Stachler et al., 2015), but the reason for the other interactions is not yet clear.

**FIGURE 3:**
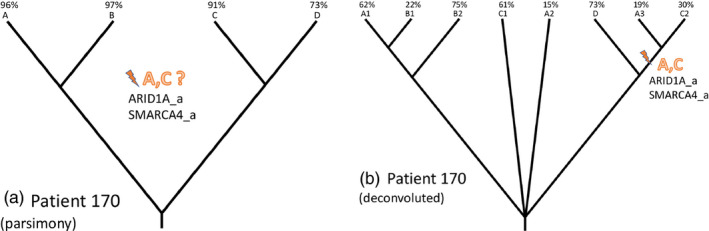
Bulk‐sample phylogeny fails to explain candidate‐locus mutations. (a) Bulk‐sample parsimony phylogeny for Patient #170. Mutations in *ARID1A* and *SMARCA4* shared by samples A and C do not fit on the phylogeny. (b) Deconvoluted phylogeny for patient #170. The mutations shared by A and C arise from a minority subclone present in both samples

We had previously made bulk‐epithelium parsimony phylogenies of these data (unpublished). In contrasting them with the deconvoluted phylogenies we noted several cases in which candidate‐locus mutations were discordant with the bulk‐epithelium phylogeny, but concordant with the deconvoluted phylogeny. An example is shown in Figure 3: In this patient, *ARID1A* and *SMARCA4* mutations arose in a subclone which gave rise to parts of samples A and C, but was not captured in the bulk‐epithelium phylogeny. This shows the importance of deconvolution in correctly evaluating the distribution of candidate mutations.

## 4 DISCUSSION

**FIGURE 4:**
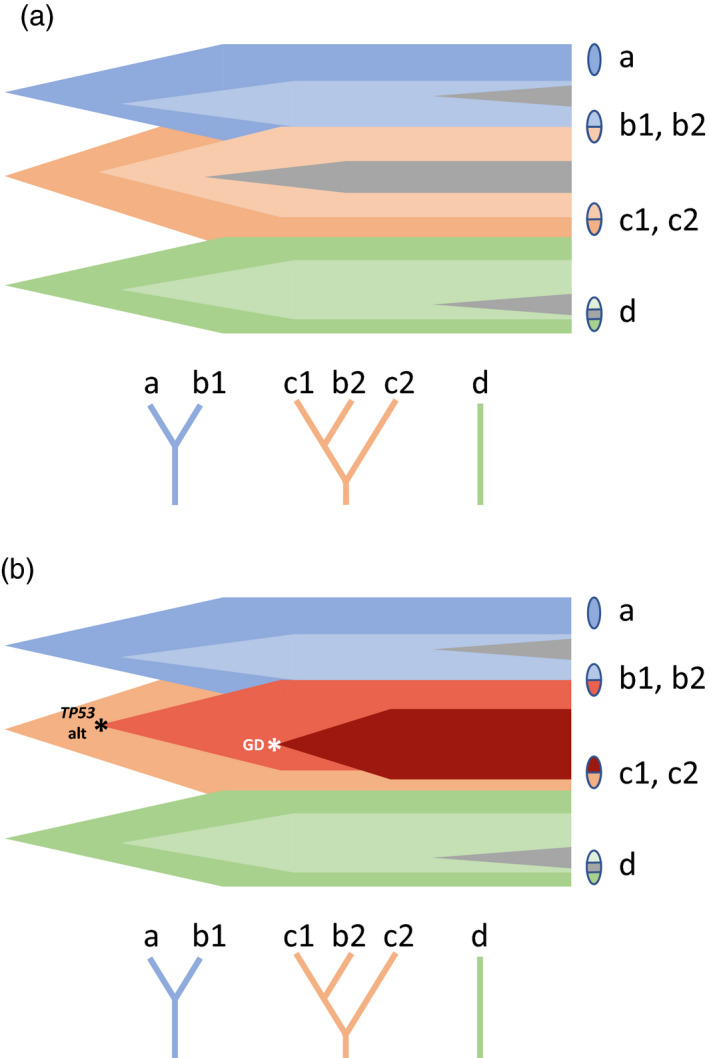
Hypothetical schematic of the history of the BE segment in NCO (panel a) and CO (panel b). In both NCO and CO, an initial expansion of lineages is followed by a period of relative stasis. In NCO, however, an early mutation in *TP53* creates a risky lineage which later undergoes genomic destabilization and genome doubling, and progresses to EA. Ovals on the left of each figure show biopsies; trees below each figure show the history of these biopsies, indicating in particular how biopsy b contains lineages from two different lines of descent

We propose a two‐phase model of the development of BE, shown schematically in Figure 4. In the first phase, one or more BE lineages arise and spread throughout the lower esophagus. During this period, there is positive selection for homozygous mutation in *CDKN2A* and *TP53*, and lineages with such mutations, if they arise, will spread across a disproportionate share of the segment. In contrast, mutations in *ARID1A* and *SMARCA4*, while they are believed to be positively selected in BE overall based on an excess of functional mutations over expectations, do not achieve large‐scale spreading; we posit that their selective advantage does not apply during initial expansion and that *SMARCA4* mutations may in fact be disadvantageous during this phase.

In the second phase, the spatial layout of the BE segment stabilizes in both CO and NCO: Geographically proximate biopsies tend to contain genetically proximate lineages even when sampled several years apart, whereas there is no tendency for samples taken at the same time to be particularly related. A similar finding was reported by Ross‐Innes et al. (2015) in a detailed study of one patient over time. We posit that even a selective advantage does not allow a lineage to spread very far once overall segment growth has ended; it may encounter difficulty displacing neighboring lineages (Kostadinov et al., 2013). This is shown by the failure of many *TP53* and *CDKN2A* mutant lineages to colonize the whole esophagus, even at the last sampling time. During this period, *ARID1A* and *SMARCA4* presumably show a survival or growth advantage resulting in an excess of functional mutations, but without long‐range spread of mutant clones.

The crypt structure of intestinal epithelium, which BE somewhat resembles, has been proposed (Cairns, 1975) to restrain the spread of mutant lineages: Stem cells are sequestered at the base of crypts and cannot colonize neighboring crypts, so expansion requires crypt fission and displacement of neighboring crypts. The replacement of squamous tissue by BE may thus represent an adaptive, if not always successful, mechanism to prevent or delay cancer in a high‐mutation environment or to otherwise manage tissue damage (Orlando, 2006).

In CO, we posit that one or more risky lineages, typically marked by *TP53* mutations, exist in the static segment—likely because they arose during initial expansion. These lineages undergo chromosomal instability, copy number variation and structural alterations, and genome doubling, and there is local expansion of the unstable clones, perhaps via colonization during wound repair in the epithelial layer. Even for such lineages, there is not widespread expansion; lineages with GD do not occupy a disproportionate share of the segment in patients who possess them, compared to a neutral mutation. Unfortunately, one of these lineages may eventually "solve" the problem of clonal expansion by invasion into the underlying stromal tissue as a tumor.

A meta‐analysis (Visrodia et al., 2016) indicated that 25% of EAs are diagnosed within one year of diagnosis of BE. These have often been interpreted as EAs which were already present but were missed by endoscopy; however, they can also be taken as evidence that the development of EA from a pre‐existing genomically unstable lineage is a rapid process. As described by Martinez et al. (2016), some proportion of BE segments are “born to be bad.” These observations fit well with our finding of a two‐phase natural history of the BE segment, with most clonal expansion occurring prior to initial diagnosis. Our model may also help explain why PPI treatment has not been clearly successful in reducing EA risk: preventing further mutational damage does not remedy the instability of existing *TP53* clones, and while it may help prevent novel *TP53* mutations, late‐arising mutations likely encounter difficulty in expanding and may incur less risk of progression than earlier ones.

There is a striking contrast between the behavior of mutations in *TP53* and *CDKN2A*. Both are selectively favored and clones which have lost the wild‐type allele tend to expand over large portions of the segment, presumably during the initial expansion phase. But lineages with mutation in *TP53* go on to chromosomal instability and cancer, whereas lineages with mutation in *CDKN2A* confer no increased risk of cancer (Li et al., 2014). There is a need to distinguish between mutations which allow a cell lineage in an expanding tissue to grow more rapidly than its neighbors—which may be entirely benign—from mutations which dysregulate growth and thereby lead to cancer (Kuhner et al., 2016).

In addition to illuminating the history of BE, our study demonstrates the importance of deconvolution and within‐patient phylogenetic analysis. In these data, bulk‐epithelium analyses undercount quasi‐independent origins of BE, understate the prevalence of homozygous candidate‐locus mutations, and (as shown in Figure 3) can suggest that candidate‐locus mutations arose multiple times when instead they were present in a minority subclone. Furthermore, even with the very limited spatial resolution of 4–6 samples per patient, phylogenetic analysis reveals spatial structure and large‐scale selective expansions. It was also apparent in working through the phylogenies by hand that copy number calling cannot reliably succeed without deconvolution, as subclonal copy number variation and subclonal ploidy variation are abundant in BE, and insisting on a bulk‐epithelium integer copy number call cannot capture the reality of such data.

Further insight will be gained by phylogenetic analysis of larger numbers of samples per patient, when such data become available. This will need to be coupled with methodological improvements: automation of the hand deconvolution procedure, and integration of copy number calling with deconvolution to avoid copy number errors induced by subclonality, especially subclones of varying ploidy. Fully automated deconvolution is a challenging methodological problem (see the review by Schwarz & Schaffer, 2017), but the data‐driven approach shown in this study may provide a basis for improving existing algorithms. The phylogenetic strategy we have used with Barrett's Esophagus is also applicable to other conditions in which multiple samples can be obtained from the same tumor or neoplasm, although it will be difficult to carry out on samples of low purity.

## CONFLICTS OF INTEREST

None declared.

## Figures and Tables

**FIGURE A1:**
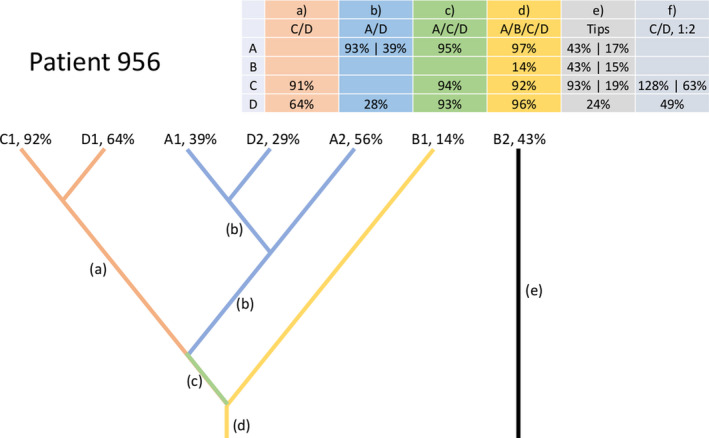
Deconvolution and phylogeny inference of Patient 956. Inset table shows VAF*2 values for each partition, labeled with upper‐case letters indicating which samples were involved; lower‐case letters show correspondence between VAF*2 values in the table and branches in the phylogeny. When two VAF*2 values are given they represent two separate peaks in the VAF histogram. In the phylogeny, tip labels indicate sample (letter), subclone (digit), and CF (percentage). Colors are used to clarify which portion of the phylogeny is drawn at each step. Branch lengths on the diagram are arbitrary

## Data Availability

All sequencing and SNP array data are under embargo until September 30, 2020, and will subsequently be available at: http://www.ncbi.nlm.nih.gov/projects/gap/cgi-bin/study.cgi?study_id=phs001912.v1.p1 Deconvoluted phylogenies are available in the Dryad repository, doi: 10.5601/dryad.866t1g1p1

## Appendix A Deconvolution procedure

We assume that SNVs have already been called and the VAF of each SNV has been estimated from read counts. We also assume that local copy number calls are available, although we acknowledge that these calls may be systematically wrong for biopsies with substantial subclonality, especially if the subclones differ in ploidy. Given these inputs, we first process the VAF values to detect clusters of co‐inherited SNVs with similar VAFs, as follows:

- Divide the SNVs into partitions, where all SNVs in a partition are shared by the same group of samples. At this step, SNVs for which one or more samples lacking the SNV have a deletion of one haplotype at the SNV’s location are discarded, as it is difficult to determine whether the SNV was present or absent prior to the deletion. We also discard SNVs in regions without a corresponding copy number call and ones located on the X or Y chromosomes. (The latter could in principle be used, but we wished to maintain consistency with Paulson et al. (in preparation), which did not use sex chromosome SNVs.)
- For each sample, divide partitions involving this sample into groups based on the local copy number call (for example, separating a group of SNVs found in 1,1 regions from another group found in 2,3 regions).
- Identify peaks in the VAF distribution for each group, using custom kernel‐based curve smoothing.
- Estimate the number of SNVs associated with each peak using the *pymix* library (Georgi et al., 2010). We treated the peaks as Gaussians with initial means at the points chosen by the kernel‐based smoothing code, and initial standard deviations set at the standard deviation of the full data for this group; the standard deviation is an overestimate if there are multiple peaks, but this did not seem to impact performance based on visual inspection. At this step, peaks associated with fewer than 100 SNVs are discarded, as are entire groups if none of their peaks contains 100+ SNVs. We did not use the *pymix* estimates of peak means, as near the boundaries of the VAF distribution space we found its peak estimates to be strongly biased away from the boundary due to violation of the Gaussian assumption; however, this problem did not seem to strongly affect estimates of the number of SNVs per peak based on visual inspection of the VAF histograms.

Custom Python scripts to carry out these steps are online at https://github.com/kuhnerlab/subclone_deconvolution.

This process yields a spreadsheet which categorizes each group of SNVs in terms of the samples which share it, its local copy number in each sample, the peak or peaks of its VAF distribution, and the number of SNVs assigned to each peak. This is the raw material for hand deconvolution and phylogeny inference. Estimating cell fractions and inferring the subclone phylogeny are done as part of the same process, as each informs the other. The peak VAF values are used to make estimates of the cell fraction (CF) contributed to each sample by the branch on which this group of SNVs arose, taking local copy number into account.

Two general rules used in deconvolution should be mentioned. First, when two VAF*2 peak values were within 10 percentage points of each other, they were considered to plausibly represent estimates of the same value, and when they were outside this range, they were not. The 10 point cutoff is arbitrary as the process generating VAF peaks is complex and resists statistical modeling. We observed that cutoffs tighter than 10 percentage points generated much more phylogenetic inconsistency. Second, we did not use groups of less than 100 SNVs for any purpose, even tie‐breaking. When we examined such small groups, we found that they often contradicted the phylogenetic signal of the larger groups and that their VAF peak estimates appeared unstable. We therefore considered them too noisy for use.

Our deconvolution algorithm relies primarily on SNVs shared by two or more samples. Private SNVs are much more difficult to interpret and are used mainly for validation. Thus, a leaf of the phylogeny represents cells that shared a common ancestor at the point of divergence from the remainder of the phylogeny, and does not attempt to account for further, private splitting of the lineage after its divergence.

To construct the phylogeny, we consider partitions in turn, starting with partitions of two samples and working toward larger partitions. We will illustrate with deconvolution of patient 956, labeling the four samples arbitrarily A, B, C, and D. Initially, we will focus on regions with a copy number call of (1,1) (normal diploid), considering other calls later; this was our approach except for samples where there were no usable (1,1) calls (generally genome doubled samples). This process is illustrated in Figure A1: Lower‐case letters in the figure correspond to the steps below.

- The most numerous partition (over 3,000 SNVs) was shared by samples C and D, with VAF*2 of 91% in C and 64% in D. We draw a clade connecting C and D. Final CF estimates will be made by a weighted average of all relevant groups, but we can add preliminary estimates now as a guide to further clade addition.
- The next most numerous partition connected A and D with a “split” in A of 93% and 39%, and 28% in D. We interpret this as the presence in A of two lineages both related to D, but with a different branching time. The SNVs with VAF*2 of 39% represent the inner tip A1, and those with VAF*2 of 93% represent the sum of the two tips A1 and A2. (We do not see VAF*2 values reflecting the outer tip A2 only, because there is no branch on which mutations shared by A2 and D, but not A1, could arise.) We also note that the D lineage seen here cannot be the one previously seen, as that lineage (D1) was related to C, whereas this one (D2) is related to A. As a consistency check, this interpretation requires that the inferred CFs of D1 and D2 not add to more than 100%; in fact, they add to 92%.
- The next partition unites A + C + D with VAF*2 of 95%, 94%, and 93%, respectively. These values clearly cannot represent additional novel lineages within A, C, or D, as the sum of inferred CFs for each sample would be far above 100%. Therefore, we connect the two subtrees previously drawn. This generates additional estimates of the CF at each tip. We check to see whether this joining generates any inferred CF more than 10 percentage points higher than tipward estimates, which would suggest an additional lineage, but it does not.
- The next partition unites A + B + C + D with VAFs of 97%, 14%, 92%, and 96%, respectively. We attach B to the A + C + D group.
- We now check private mutations to see whether their VAF*2 values contradict the inferred tree; for example, observing private VAF*2 values much greater than 56% in sample A would be a contradiction, as we are interpreting A as two separate lineages with CFs of 56% and 39%, and separate lineages should not share substantial private SNVs. In this case, the VAF*2 peak for sample A is split 43% and 17%. This illustrates the difficulty with private SNVs: It is not clear whether these private SNVs arose on A1, A2, or both, nor how many times these lineages split after separating from the main tree. We therefore do not try to assign SNVs to A1 and A2, but simply note that the values are compatible with the tree.
  Checking private SNVs in the other tips, we find B split with 43% and 15%. While the SNVs with VAF*2 of 15% could plausibly have arisen on the B branch in our phylogeny, those with VAF*2 of 43% could not, as the branch has inferred CF of only 14%. (Mismatches of up to 10 percentage points were allowed.) We therefore draw a detached B2 branch with inferred CF of 43%, representing a separate origin. This is the only circumstance under which we draw branches based purely on private SNVs.
  Not all of sample B seems to be accounted for (43%+14%=57%). There are several possibilities for the rest of the sample. The tip labeled B2 might confound two separate lineages with CF near 43%. Alternatively, the remainder of B might be non‐BE tissue (or BE tissue with very few mutations), or might consist of multiple lineages each with CF below the detection threshold of approximately 15%; such lineages could arise anywhere in the phylogeny, or represent further independent origins. Without a reliable way to distinguish these possibilities, we did not base lineages on such failures to explain all cells in a sample. In this case, 90%+ of each of the other samples is accounted for, which is consistent with the expected purity of epithelial isolated BE tissues of around 95%.
  Sample C's private mutations have a split VAF*2 of 93% and 19%. These are compatible with the inferred CFs. Since the VAF*2 of 93% is higher than previous estimates of the CF for leaf C1, we include these private SNVs in the estimate. The lower VAF*2 is compatible with leaf C2, but these could also represent a split of C1; we therefore do not attempt to assign them to a specific leaf, and do not use them in any CF calculations. Similarly, sample D's private mutations have a VAF*2 of 24%, which is compatible with both cell fractions assigned to the D tips.
- We then check groups with copy number calls other than (1,1). The C + D partition has SNVs falling into regions of (1,2) call in C and D. VAF*2 in C was split 128% and 63%; in D, a similar split was seen, but only the lower value of 49% had enough SNVs to meet the threshold. Splits are expected for a (1,2) call: Mutations which arose on the duplicated haplotype before the duplication event will be present as 2/3 of total copies in each cell, whereas mutations which arose on the nonduplicated haplotype, or after the duplication on one of the duplicated haplotypes, will be present as 1/3 of total copies. We therefore interpret the VAF (not VAF*2) values accordingly; the higher VAF is 2/3 CF, and the lower is 1/3 CF. This gives CF estimates of 94.5% for both splits of C, and 73% for D, which are within tolerances of the CF estimates from (1,1) partitions (92% and 64%, respectively). Elsewhere in the tree, the number of SNVs in a given nondiploid copy call did not reach the 100 + SNV threshold.

We make final estimates of CF for each leaf by averaging all relevant estimates, weighted by the number of SNVs contributing to each. Private SNVs contribute to this estimate only in two circumstances: (1) when a tip's existence is known only due to private SNVs, such as B2 in this patient; (2) when the inferred CF of the private SNVs was higher than (but not incompatible with) the CF for their leaf, in which case they were taken to represent an additional estimate of the CF, as with C1 in this patient. When the inferred CF was not higher we did not use it, as it may reflect lossage due to splits subsequent to the lineage's divergence from the tree.

A noteworthy point for this patient is that our inference process indicates a separate origin of B2: While it must, of course, have a common ancestor with other tissues in the patient, there were less than 100 SNVs inherited from the common ancestor, suggesting it did not yet have the typical high BE point mutation rate. Previously, this patient had been presumed to have a single origin due to many SNVs shared among all four samples (due to B1). Only phylogenetic deconvolution revealed the second origin.

About 1/3 of patients could be straightforwardly analyzed in this way. Complications arose in the others: We will go through each complication in turn.

### Decreasing CF toward the tips

In many cases, the proportion of a leaf inheriting down a particular lineage could be estimated at multiple levels in the phylogeny: In our example patient, the C + D, A + C + D, and A + B + C + D partitions are all inferred to involve the same C and D lineages, and thus, each provides a separate estimate of the C and D CF’s. As long as these estimates were within 10 percentage points in either direction, we accepted them as estimates of the same quantity. When the estimates increased by more than 10 percentage points moving toward the tips of the tree, this was taken as a logical inconsistency in need of resolution: There is no biological way for a sample to inherit 80% of its cells from a parent lineage, but only 50% from that parent's parent.

However, CF estimates that decrease moving from root to tip were interpreted as the splitting off of additional lineages. For example, if A + C + D had accounted for 80% of sample C, but C + D accounted for only 50%, we assumed that a lineage accounting for the remaining 30% of cells in C diverged from the A + C + D lineage prior to divergence of A. Since such a lineage is inferred, not from a specific group of SNVs, but from a decrease in the VAF of SNVs in C + D compared to A + C + D, we do not know whether it actually represents a single lineage or more than one, but we conservatively draw it as a single lineage, in accordance with our decision not to account for splitting in leaves.

### Ambiguity

We sometimes observed a pattern in which a partition of two samples (for example A and B) showed splits in both of them. When GD was not available as an explanation (see below), we interpreted this as evidence for a branch ancestral to A + B which has split twice, yielding two A and two B lineages. However, the splits themselves do not indicate whether the outgroup of this clade was an A lineage or a B lineage.

For most of these situations, it was possible to resolve whether A or B split off first by comparing the VAFs of the shared mutations. As illustrated in Figure A2, if one B and two A lineages descend from a common ancestor (panel a), the VAFs of mutations that arise on the intermediate branch of the tree will be high in A, and low in B. No branch will yield mutation VAFs that are high in B but low in A. In the reverse case (panel b), if two B and one A lineages descend from a common ancestor, the VAFs of the mutations that arise on the intermediate branch of the tree will be low in A, and high in B, with no branch yielding high‐A and low‐B mutation VAFs. Therefore, by classifying each shared mutation as “high” or “low” for both samples, we can determine that the first scenario is more likely if there are many more “high A, low B” mutations than “high B, low A” mutations, and vice versa.

A third possibility also exists: if the two As have similar CFs, and the two Bs also have similar CFs, the phylogeny shown in panel (c) is possible. In this case, you would see very few mutations that were high in one sample and low in the other: all mutations would instead be low in both or high in both. However, this situation is impossible to distinguish from one of the previous scenarios where the branch between A2 and B2 is very short. In such cases, we drew the tree as a polytomy (panel d), having no basis to prefer one resolution over the other.

We also drew polytomies where partitioning in a group of 3 or more lineages did not uniquely identify the branching order. For example, if we found an A + B + C partition but none of A + B, A + C, or B + C reached the 100 + SNV cutoff, we drew this as an A + B + C polytomy.

### Genome doubling

We used previous estimates of which samples were genome doubled, with the caveat that existing ploidy estimation assumes that a sample has uniform ploidy throughout, which is not necessarily the case.

When‐genome doubling happens on a particular branch of a phylogeny, it will sometimes be possible to detect a factor‐of‐two relationship between splits in all of the descendant lineages. This arises because mutations which occur before genome doubling, and thus double along with it, have twice the VAF of those that occur later; a GD event midway through a branch thus produces two VAF*2 values differing by a factor of two. The pattern will not always be visible, however; if the GD occurs very early in the branch, mutations arising before it may be too scarce for analysis, and similarly for those arising after a late GD. We used these doubling patterns to infer the branch or branches on which GD arose. We used the minimum number of GD events to explain all putative GD samples, given the assumption that GD does not revert to diploidy. In four cases, this led to the inference that GD had happened more than once.

In two patients, we saw a doubling pattern consistent with GD, but there was no external evidence for GD in those samples. We assumed that the factor‐of‐two relationship was coincidental rather than assuming undetected GD. In one patient, 997, our data disagreed strongly with the previous assessment that two samples were genome doubled and two were not. We consulted with the team that made the initial ploidy calls and found that they had been divided on whether two samples or all four were GD, so for this study we accepted the data‐driven inference of GD in all samples for 997. This was the only time we overruled the assignments of genome doubling from Paulson et al. (in preparation).

### Spatial clustering of a partition

Normally, the 100 + SNVs of a phylogenetic partition were dispersed across the genome. In a few cases, they were instead strongly concentrated on one or a few chromosomes. We believe that a partition of this kind represents large deletions or losses of heterozygosity missed by the copy number calling procedure. The real phylogenetic partition includes one or more additional samples, but in the regions of clustering those samples have lost the SNV‐bearing haplotype. We therefore dropped such partitions from analysis, consistent with our handling of partitions with a known deletion.

### Allelic dropout

Phylogenetic deconvolution relies on being able to accurately determine which samples possess a given SNV and which do not. However, a mutation may be missed in a sample by chance. When this happens randomly, for example due to low local read depth, it is unlikely to create a group of 100 + SNVs all of which are misleading. However, when one sample in a partition is at low CF, missed SNVs in that specific sample can become frequent and form a detectable false partition.

We initially attempted to calculate the expected number of dropouts based on local read depth and assuming a binomial distribution of reads. However, this value was consistently lower than the number of putative dropout SNVs we observed, probably because we were not able to model the best‐of‐three mutation calling strategy accurately. We therefore treated a partition as a dropout whenever (a) it failed to fit the phylogeny created by other partitions, and (b) it could be made to fit by assuming by dropout of one or (rarely) more low‐CF samples, where low CF was defined as <30%, from an existing partition. When we could find such a solution, we treated the SNVs as if they belonged to the partition including the additional sample(s). In a small number of cases, we could not reach a consistent phylogeny unless we assumed dropout from a CF greater than 30%; we still accepted the deconvolution if it appeared otherwise coherent.

In an attempt to reduce the problem of allelic dropout, we tested a procedure in which, once an SNV had been identified as present in a patient (based on 2+ callers as usual), it was scored as present in a biopsy if at least 2 mutant reads were seen, even if they were not called by the mutation callers. While this reduced dropout, in initial examination of several patients it also generated numerous low‐VAF partitions which did not fit the inferred phylogeny and were apparently spurious. We therefore rejected this approach.

### Local copy number

We initially intended to use information from all inferred copy number states as input to our estimates of CF. However, in examining multiple patients who had both (0,1) and (0,2) calls, we found that the (0,1) calls often indicated a substantially different CF than the (0,2), and that the (0,2) were more consistent with CF estimates from other calls such as (1,1). We believe this happens because local copy number is generally not homogeneous among all cells in a biopsy, due to both subclonality and non‐BE cells. Wild‐type cells have twice as many haplotypes to contribute as cells with a (0,1) deletion state, and even a small number of these throws off calculation of the expected CF. Since we were not, in general, able to assign copy number events to specific lineages in the tree, we were not able to make a more accurate conversion between observed VAF and inferred CF. We therefore chose to disregard (0,1) calls. The problem was much less marked for calls such as (1,2), as the ratio between CNV cells and wild‐type cells was less extreme.

In most cases, unbalanced calls such as (1,2) yielded splits in the SNV distribution, as shown in the detailed example in Figure A1. However, fairly frequently we would find a group with a (1,2) call and only a single VAF*2 value; furthermore, the single value would be consistent with the CF inferred from (1,1) or (2,2) calls and not with our expectations for (1,2). We believe there are two ways this situation can arise. If the duplication which leads to the (1,2) call is in a subclone other than the one whose SNVs are currently being assessed, we would not expect a split and would expect VAF*2 to be fairly close to CF. Alternatively, we have found that if a region is (1,1) in one subclone and (2,2) in another within the same sample, it is impossible for *pASCAT* to make a satisfactory integer resolution of the copy numbers, and (1,2) is sometimes assigned despite lack of evidence for allelic imbalance. This was verified with the checking program *CNValidator* (Smith et al., 2019) for a subset of calls in which the local haplotype could be resolved. In either case, when an apparently imbalanced call yielded no split and appeared consistent with the CF estimate from other groups, we accepted it as such despite the copy number call.

In a few cases, we could not agree on any interpretation of a group with nondiploid copy number, especially for unbalanced calls. Such groups were considered in drawing the phylogeny (as the presence of shared SNVs among particular samples is evidence of a relationship among them, no matter what the VAF) but were not used for inference of CF. This problem was most prominent in patients with a mixture of GD and non‐GD lineages, for whom the copy number calls were particularly likely to be misleading.

### Failures

In two CO patients (74 and 396), we were unable to reach any confidence in our deconvolution. Both patients had GD in some samples but not others and apparent high levels of subclonality. In these patients, multiple phylogenetically incompatible partitions were observed which could not be cleanly explained with dropouts. We have omitted these two patients from all analyses. As these patients were likely at the extreme end of subclonal complexity, our estimates of subclonal prevalence will be biased downwards.

### Computation of final CF estimates

We computed CF estimates to the nearest integer by doing a grid search among possible CFs for all lineages attributed to a particular sample, constraining the sum of CFs for any single sample to be <=100, and choosing the inferred CFs that minimized the sum of squared differences between the estimates (weighted by the number of SNVs contributing to each) and the inferred value. In a small number of cases this led to lineages being assigned a CF so low that we would not have expected to detect the lineage. Lacking a better alternative, we let these estimates stand.

## Appendix B Detection of multiple putative origins

It was not intuitive to us that multiple origins could be detected even when all samples from a patient shared SNVs, but in several patients this proved to be the case: We give one example here to illustrate the logic.

Patient #266 had four samples which we will refer to as A‐D for convenience. A group of SNVs were found in all four samples, with the following inferred CF (twice the VAF): A, 93%; B, 95%; C, 18%; D, 89%. (All CFs were estimated only from areas inferred to have normal copy number.) The high values for A, B, and D suggest that the vast majority of cells in these samples arose from a lineage ancestral to all four samples; however, only a minority of sample C represents this lineage.

Sample C had private SNVs with inferred CFs of 89% and 23%. The SNVs with inferred CF of 23% may represent the same lineage as the 18% shared ancestral SNVs, as these numbers are within tolerance of each other (we allowed discrepancies of up to 10 percentage points). However, the SNVs with inferred CF of 89% must represent a separate lineage. As there were no groups of mutations shared between C and any other sample at such high CF, we infer that these mutations arose in a lineage which was ancestral to C and to no other sample. Thus, there are two apparently independent BE clades in this patient: one which was ancestral to A, B, and D as well as a minority clone of C, and one which was ancestral to the majority of C.

## References

[eva13125-bib-0001] Benjamini, Y. , & Hochberg, Y. (1995). Controlling the false discovery rate: A practical and powerful approach to multiple testing. Journal of the Royal Statistical Society. Series B, Statistical Methodology, 57, 289–300.

[eva13125-bib-0002] Cairns, J. (1975). Mutation selection and the natural history of cancer. Nature, 255, 197–200. 10.1038/255197a0 1143315

[eva13125-bib-0003] Cibulskis, K. , Lawrence, M. S. , Carter, S. L. , Sivachenko, A. , Jaffe, D. , Sougnez, C. , … Getz, G. (2013). Sensitive detection of somatic point mutations in impure and heterogeneous cancer samples. Nature Biotechnology, 31, 213–219. 10.1038/nbt.2514 PMC3833702 23396013

[eva13125-bib-0004] Cingolani, P. , Platts, A. , Wang, L. L. , Coon, M. , Nguyen, T. , Wang, L. , … Ruden, D. M. (2012). A program for annotating and predicting the effects of single nucleotide polymorphisms, SnpEff: SNPs in the genome of Drosophila melanogaster strain *w1118; iso‐2; iso‐3* . Fly, 6, 1–13. 10.4161/fly.19695 PMC3679285 22728672

[eva13125-bib-0005] Contino, G. , Vaughan, T. L. , Whiteman, D. , & Fitzgerald, R. C. (2017). The evolving genomic landscape of Barrett's esophagus and esophageal adenocarcinoma. Gastroenterology, 153, 657–673.e1. 10.1053/j.gastro.2017.07.007 28716721 PMC6025803

[eva13125-bib-0006] Curtius, K. , Wong, C.‐J. , Hazelton, W. D. , Kaz, A. M. , Chak, A. , Willis, J. E. , … Luebeck, E. G. (2016). A molecular clock infers heterogeneous tissue age among patients with Barrett’s esophagus. PLoS Computational Biology, 12, e1004919 10.1371/journal.pcbi.1004919 27168458 PMC4864310

[eva13125-bib-0007] Georgi, B. , Costa, I. G. , & Schliep, A. (2010). PyMix ‐ The Python mixture package ‐ a tool for clustering of heterogeneous biological data. BMC Bioinformatics, 11, 9 10.1186/1471-2105-11-9 20053276 PMC2823712

[eva13125-bib-0008] Hu, Q. , Sun, T.‐T. , Hong, J. , Fang, J.‐Y. , Xiong, H. , & Meltzer, S. J. (2017). Proton pump inhibitors do not reduce the risk of esophageal adenocarcinoma in patients with Barrett's esophagus: A systematic review and meta‐analysis. PLoS One, 12, e0169691 10.1371/journal.pone.0169691 28072858 PMC5224998

[eva13125-bib-0009] Kostadinov, R. L. , Kuhner, M. K. , Li, X. , Sanchez, C. A. , Galipeau, P. C. , Paulson, T. G. , … Maley, C. C. (2013). NSAIDS modulate clonal evolution in Barrett’s esophagus. PLOS Genetics, 9(6), e1003553 10.1371/journal.pgen.1003553 23785299 PMC3681672

[eva13125-bib-0010] Kuhner, M. K. , Kostadinov, R. , & Reid, B. J. (2016). Limitations of the driver/passenger model in cancer prevention. Cancer Prevention Research, 9, 335–338. 10.1158/1940-6207 26932841 PMC4856031

[eva13125-bib-0011] Li X. , Galipeau P. C. , Paulson T. G. , Sanchez C. A. , Arnaudo J. , Liu K. , … Reid B. J. (2014). Temporal and Spatial Evolution of Somatic Chromosomal Alterations: A Case‐Cohort Study of Barrett's Esophagus. Cancer Prevention Research, 7(1), 114–127. 10.1158/1940-6207.capr-13-0289 24253313 PMC3904552

[eva13125-bib-0012] Martincorena, I. , Raine, K. M. , Gerstung, M. , Dawson, K. J. , Haase, K. , Van Loo, P. , … Campbell, P. J. (2017). Universal patterns of selection in cancer and somatic tissues. Cell, 171, 1823 10.1016/j.cell.2017.08.042 PMC5720395 29056346

[eva13125-bib-0013] Martinez, P. , Timmer, M. R. , Lau, C. T. , Calpe, S. , Sancho‐Serra, M. D. C. , Straub, D. , … Krishnadath, K. K. (2016). Dynamic clonal equilibrium and predetermined cancer risk in Barrett’s oesophagus. Nature Communications, 7, 12158 10.1038/ncomms12158 PMC4992167 27538785

[eva13125-bib-0016] Orlando, R. C. (2006). Mucosal defense in Barrett’s Esophagus In P. Sharma , & R. Sampliner (Eds.), Barrett’s Esophagus and Esophageal Adenocarcinoma, 2nd ed. (pp. 60–72). Oxford, UK Blackwell Publishing Ltd..

[eva13125-bib-0018] Ross‐Innes, C. S. , Becq, J. , Warren, A. , Cheetham, R. K. , Northen, H. , O'Donovan, M. , … Fitzgerald, R. C. (2015). Whole‐genome sequencing provides new insights into the clonal architecture of Barrett's esophagus and esophageal adenocarcinoma. Nature Genetics, 47, 1038–1046. 10.1038/ng.3357 26192915 PMC4556068

[eva13125-bib-0019] Saunders C. T. , Wong W. S. W. , Swamy S. , Becq J. , Murray L. J. , Cheetham R. K. (2012). Strelka: accurate somatic small‐variant calling from sequenced tumor–normal sample pairs. Bioinformatics, 28(14), 1811–1817. 10.1093/bioinformatics/bts271 22581179

[eva13125-bib-0020] Schwarz, R. , & Schaffer, A. A. (2017). The evolution of tumour phylogenetics: Principles and practice. Nature Reviews Genetics, 18, 213–229. 10.1038/nrg.2016.170 PMC5886015 28190876

[eva13125-bib-0021] Seabold, S. , & Perktold, J. (2010). statsmodels: Econometric and statistical modeling with python. Proceedings of the 9th Python in Science Conference.

[eva13125-bib-0022] Shaheen, N. J. , Falk, G. W. , Iyer, P. G. , Gerson, L. B. , American College of Gastroenterology (2016). Clinical guideline: Diagnosis and management of Barrett’s esophagus. American Journal of Gastroenterology, 111, 30–50. 10.1038/ajg.2015.322 PMC10245082 26526079

[eva13125-bib-0023] Smith, L. P. , Yamato, J. A. , & Kuhner, M. K. (2019). CNValidator: Validating somatic copy‐number inference. Bioinformatics, 35, 2660–2662. 10.1093/bioinformatics/bty1022 30541069 PMC6662281

[eva13125-bib-0024] Stachler, M. D. , Taylor‐Weiner, A. , Peng, S. , McKenna, A. , Agoston, A. T. , Odze, R. D. , … Bass, A. J. (2015). Paired exome analysis of Barrett’s esophagus and adenocarcinoma. Nature Genetics, 47, 1047–1055. 10.1038/ng.3343 26192918 PMC4552571

[eva13125-bib-0025] Van Loo P. , Nordgard S. H. , Lingjaerde O. C. , Russnes H. G. , Rye I. H. , Sun W. , … Kristensen V. N. (2010). Allele‐specific copy number analysis of tumors. Proceedings of the National Academy of Sciences, 107(39), 16910–16915. 10.1073/pnas.1009843107 PMC2947907 20837533

[eva13125-bib-0026] Visrodia K. , Singh S. , Krishnamoorthi R. , Ahlquist D. A. , Wang K. K. , Iyer P. G. , & Katzka D. A. (2016). Magnitude of Missed Esophageal Adenocarcinoma After Barrett’s Esophagus Diagnosis: A Systematic Review and Meta‐analysis. Gastroenterology, 150(3), 599–607. 10.1053/j.gastro.2015.11.040 26619962 PMC4919075

[eva13125-bib-0027] Wang, X. , Ouyang, H. , Yamamoto, Y. , Kumar, P. A. , Wei, T. S. , Dagher, R. , … McKeon, F. (2011). Residual embryonic cells as precursors of a Barrett's‐like metaplasia. Cell, 145, 1023–1035. 10.1016/j.cell.2011.05.026 21703447 PMC3125107

[eva13125-bib-0028] Wilm, A. , Aw, P. P. K. , Bertrand, D. , Yeo, G. H. T. , Ong, S. H. , Wong, C. H. , … Nagarajan, N. (2012). LoFreq: A sequence‐quality aware, ultra‐sensitive variant caller for uncovering cell‐population heterogeneity from high‐throughput sequencing datasets. Nucleic Acid Research, 22, 11189–11201. 10.1093/nar/gks918 PMC3526318 23066108

